# Supplementary figures and images for: Holobiont dysbiosis or acclimatation? Shift in the microbial taxonomic diversity and functional composition of a cosmopolitan sponge subjected to chronic pollution in a Patagonian bay
Source: PeerJ. 2024 Aug 21;12:e17707. doi: 10.7717/peerj.17707 (PMC11344537; doi:10.7717/peerj.17707)

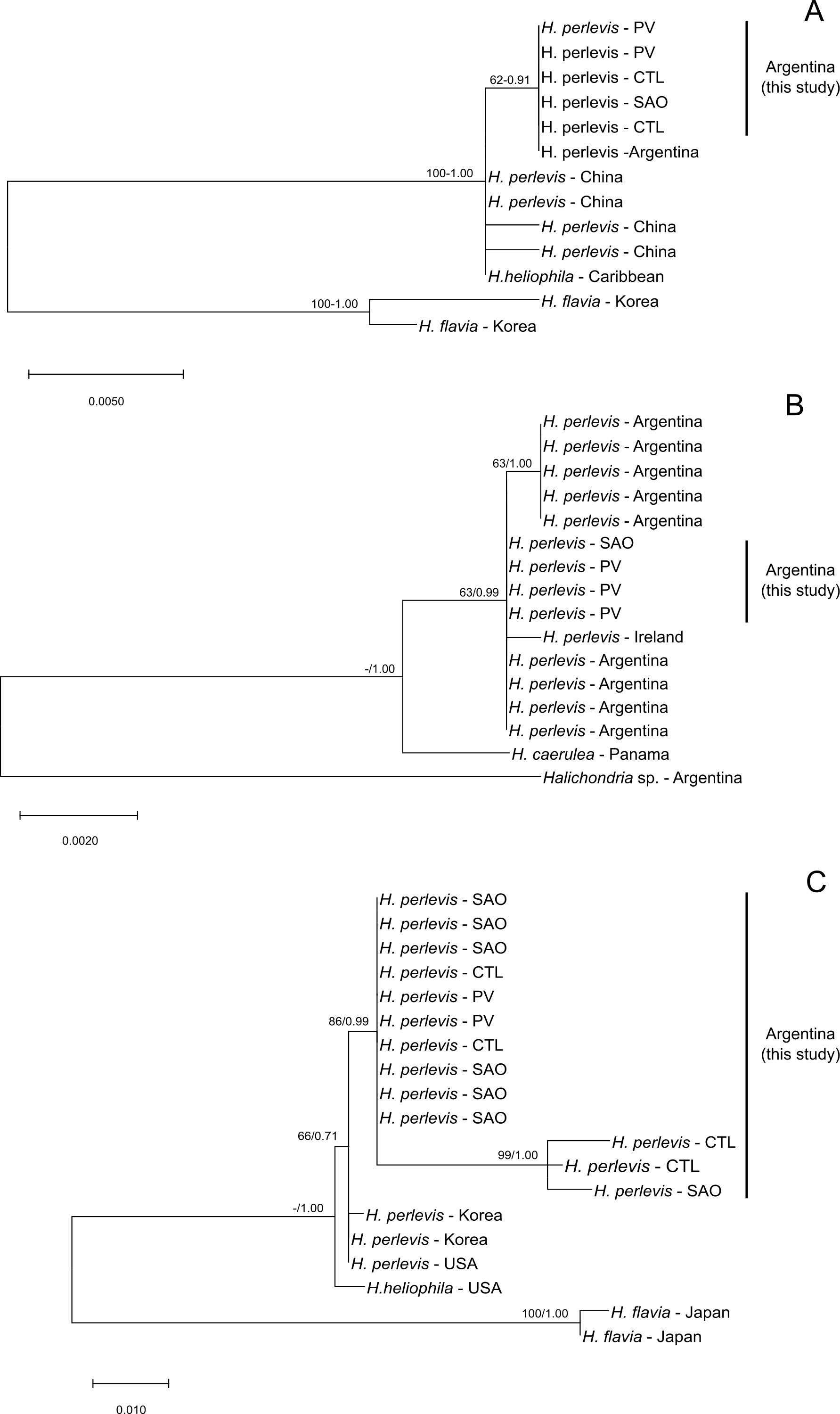

Supplement: Supplemental Information 1 — Relationships were recovered using CO1 (A), 18S (B), and ITS2 (C) sequences. Phylogenetic reconstructions recovered through the Maximum Likelihood (ML) and Bayesian methods. Numbers at nodes are Bootstrap support values (ten and thirty thousand, respectively). Hymeniacidon sinapium is presented as H. perlevis following Turner 2020 (see Table S2 for sequences references). [file peerj-12-17707-s001.png]

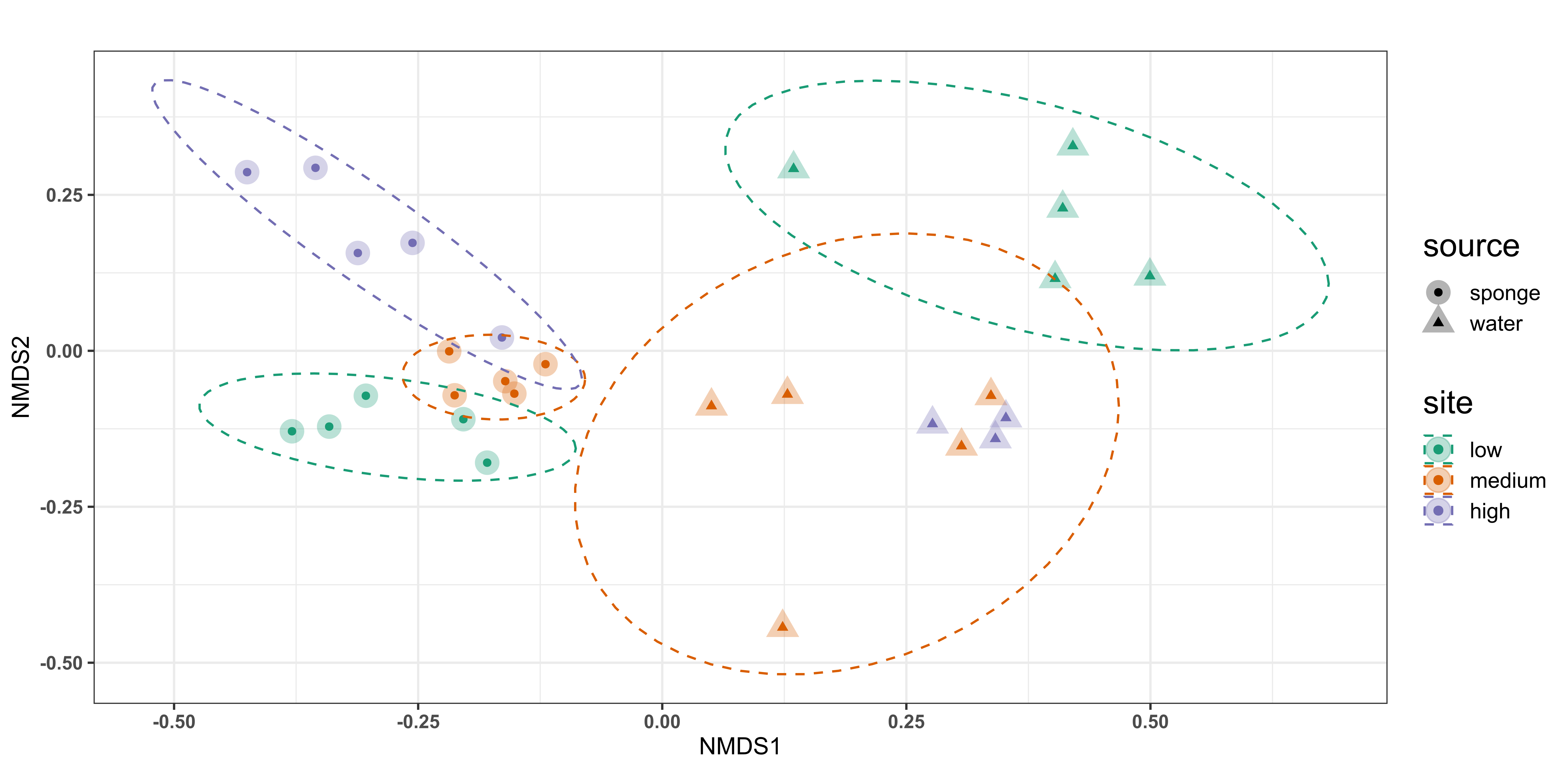

Supplement: Supplemental Information 2 — Ellipses indicate a confidence level of 80%. Ordination stress is 0.1. Low, medium, and high refer to sites with different pollution levels. The description of pollution levels can be found in Table S1. [file peerj-12-17707-s002.png]

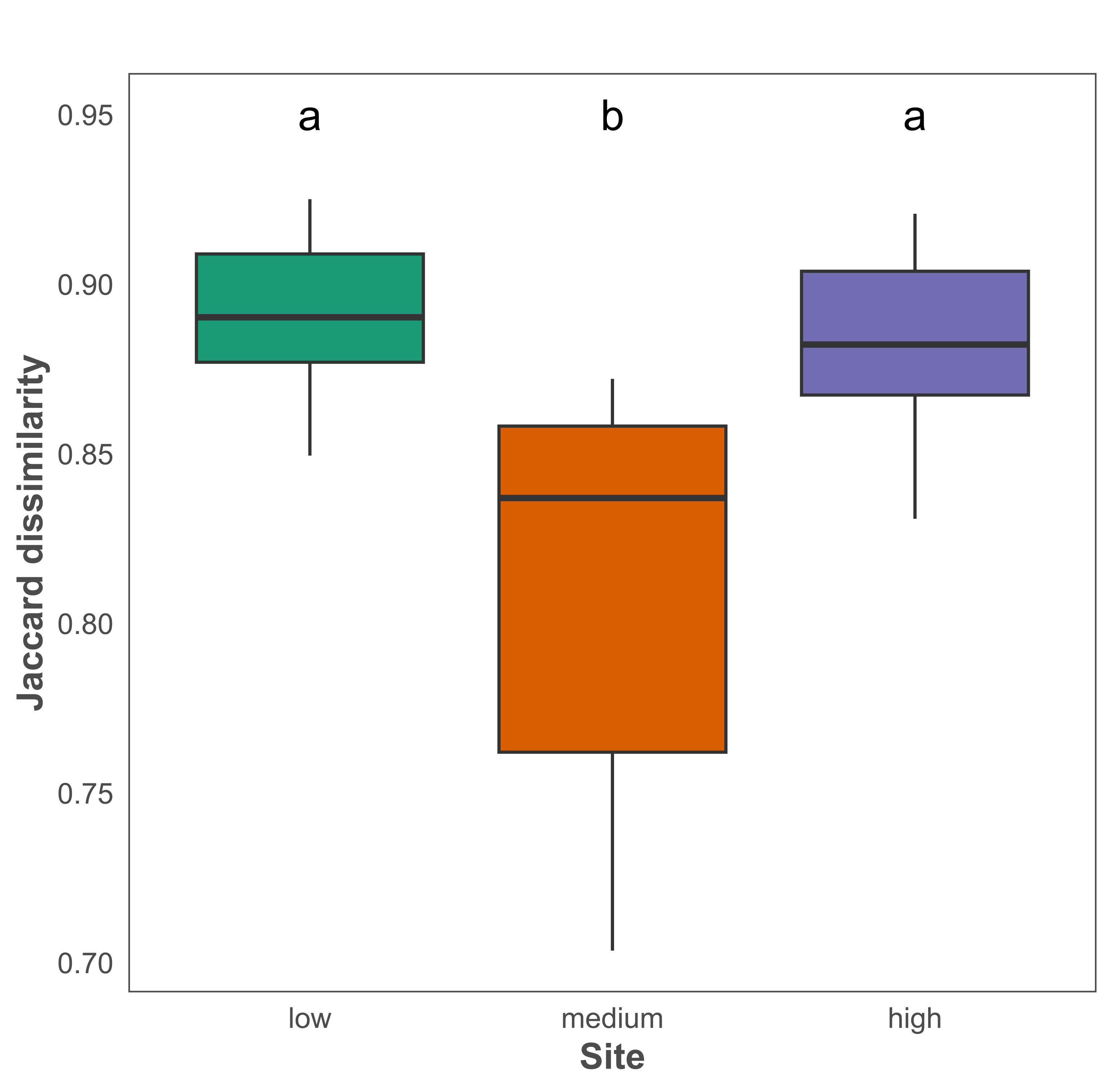

Supplement: Supplemental Information 3 — Low, medium, and high refer to sites with low, medium and high pollution. The description of pollution levels can be found in Table S1. [file peerj-12-17707-s003.png]

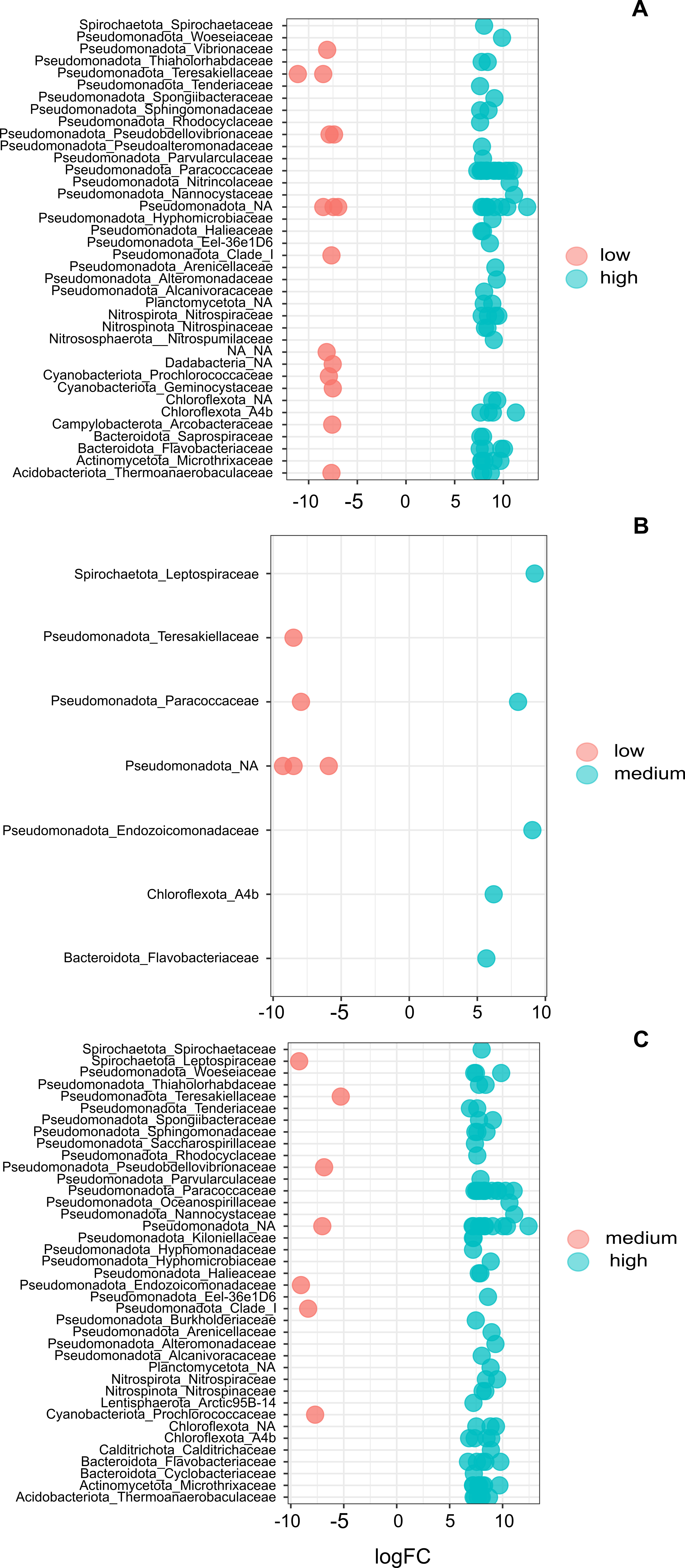

Supplement: Supplemental Information 4 — A) Microbes of sponges from sites with low- and high-pollution, B) with low- and medium-pollution, and C) with high- and medium-pollution. [file peerj-12-17707-s004.png]

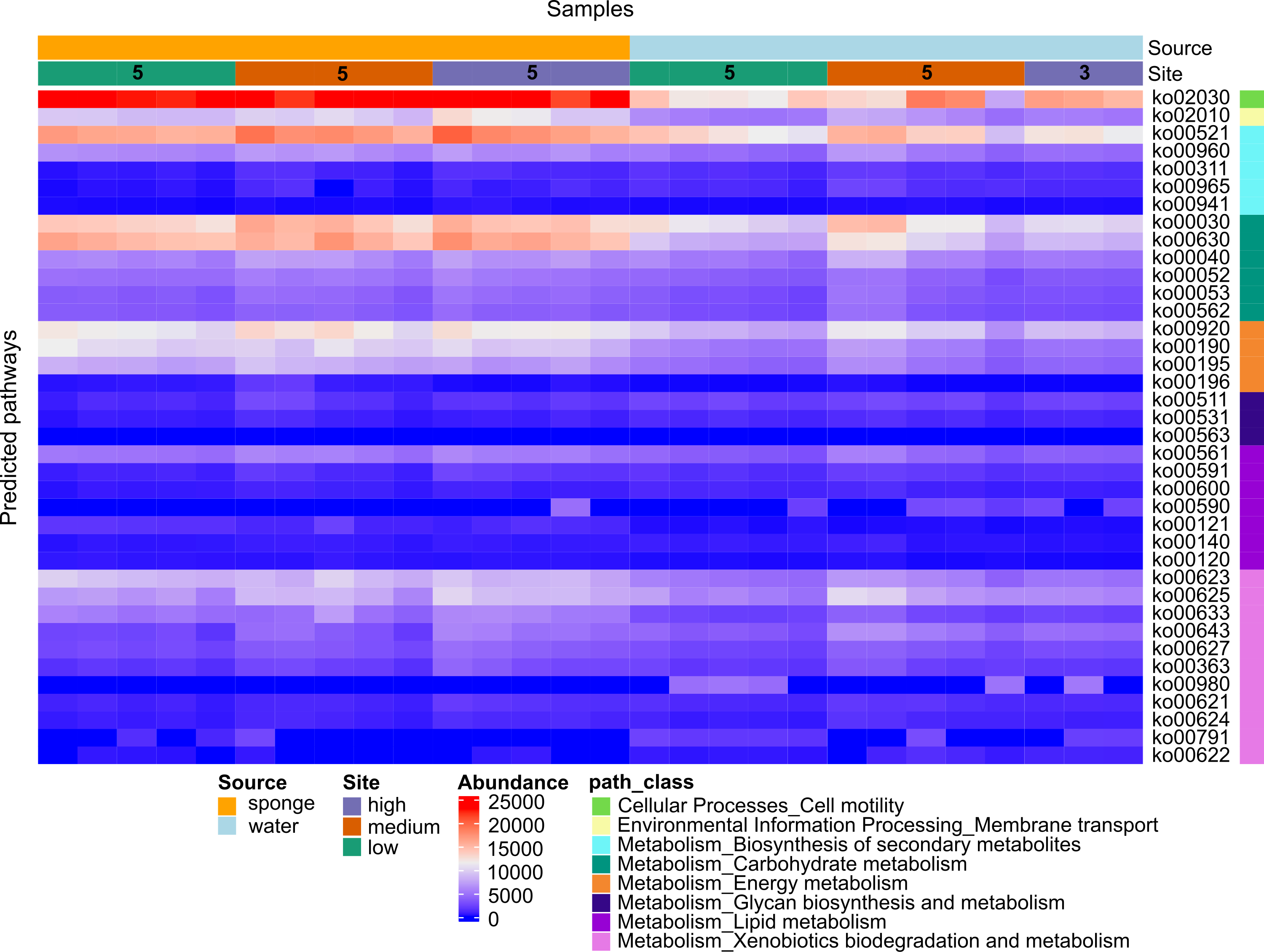

Supplement: Supplemental Information 5 — Low, medium, and high represent sites with different levels of pollution (see Table S1). Numbers in Site horizontal color bar indicate the number of samples. [file peerj-12-17707-s005.png]

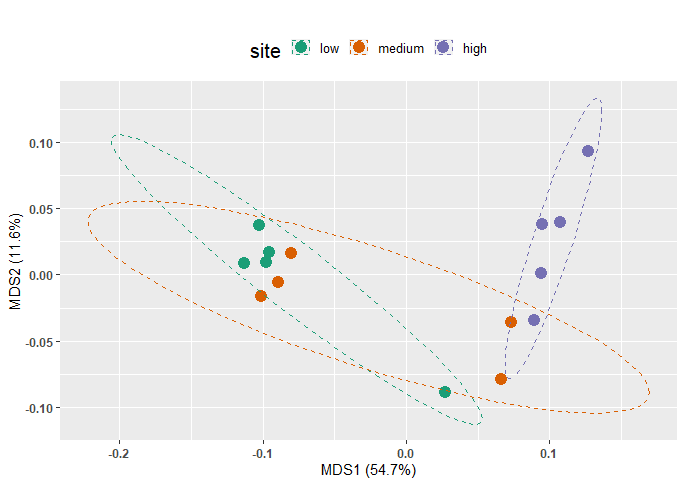

Supplement: Supplemental Information 6 — Low, medium, and high represent sites with different levels of pollution (see Table S1). Stress level=0.065. [file peerj-12-17707-s006.png]

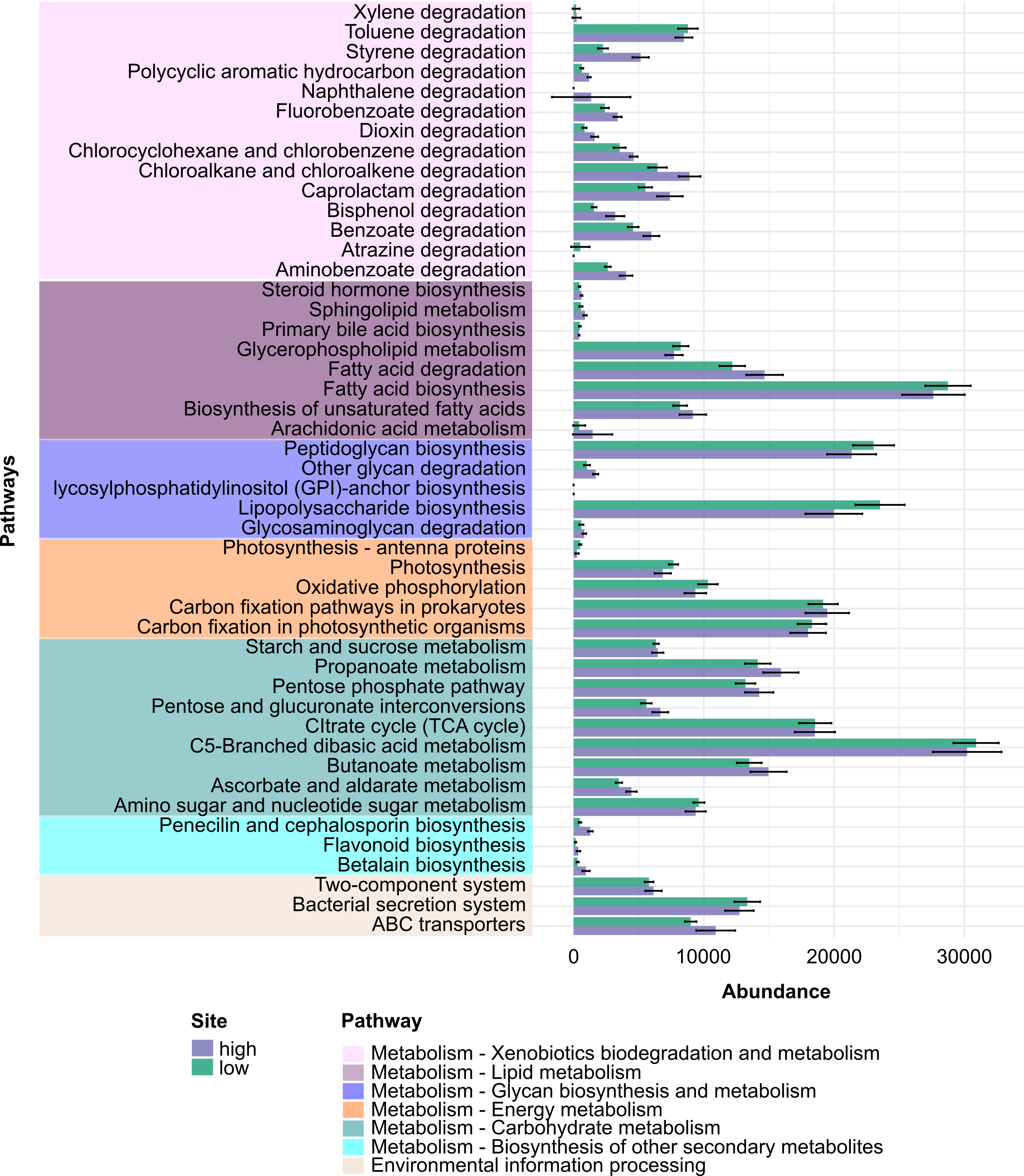

Supplement: Supplemental Information 7 — Low and high represent sites with contrasting levels of pollution (see Table S1). [file peerj-12-17707-s007.png]
