# Supplementary material for: Holobiont dysbiosis or acclimatation? Shift in the microbial taxonomic diversity and functional composition of a cosmopolitan sponge subjected to chronic pollution in a Patagonian bay
Source: PeerJ. 2024 Aug 21;12:e17707. doi: 10.7717/peerj.17707 (PMC11344537; doi:10.7717/peerj.17707)
Supplement: Supplemental Information 8 — Canada Government guidelines reference values are included for reference. ND: non-detected concentration; NA: not available data. [file peerj-12-17707-s008.docx]

# **Holobiont dysbiosis or acclimatization? Shift in the microbial taxonomic diversity and functional composition of a cosmopolitan sponge subjected to chronic pollution in a Patagonian Bay**

Marianela Gastaldi^1,2^, M. Sabrina Pankey^3^, Guillermo M. Svendsen^1,4*^, Alonso I. Medina^1^, Fausto N. Firstater^1,2^, Maite A. Narvarte^1,2^, Mariana Lozada^5^, Michael P. Lesser^3^

| Variable | high | medium | low | Reference | Guideline-  Aquatic Life | Guideline-  Recreation | Guideline-Agricultural |
| --- | --- | --- | --- | --- | --- | --- | --- |
| Nitrate (mg/L) | 62-99^1^  3.5-5.3^2^  3.7-6.8^3^  8.1-9.9^4^ | 49-74^1^  2.3^4^ | 1.5-3.5^2^  < 2.5^3^  2.5-2.7^4^ | ^1^Saad et al. 2019  ^2^Martinetto et al. 2010  ^3^Martinetto et al. 2010  ^4^Marello et al. in prep | < 16 | < 43 |  |
|  |  |  |  |  |  |  |  |
| Nitrite (mg/L) | 0.064 | ND | 0.0005 | Marello et al. in prep |  | < 4.3 |  |
|  |  |  |  |  |  |  |  |
| Ammonia (mg/L) | 0.037-1.3^1^  0.23-1.5^2^ | NA | 0.009-0.15^1^  < 0.12^2^ | ^2^Martinetto et al. 2010  ^3^Martinetto et al. 2010 | < 1.1 | - |  |
|  |  |  |  |  |  |  |  |
| Pb (mg/kg) | 13-15 | 8-10 | NA | Idaszkin et al. 2015, Marinho et al. 2016 | < 30.2 |  | < 1.4 |
|  |  |  |  |  |  |  |  |
| Zn (mg/kg) | 25-48 | 17-25 | NA | Idaszkin et al. 2015, Marinho et al. 2016 | < 124 |  | < 200 |
|  |  |  |  |  |  |  |  |
| Cu (mg/kg) | 3.5-11 | 5 | NA | Idaszkin et al. 2015, Marinho et al. 2016 | < 19 |  | < 63 |
|  |  |  |  |  |  |  |  |

Table S1: Concentration of nutrients and heavy metals pollutants reported previously for the studied sites. Canada Government guidelines reference values are included for reference. ND: non-detected concentration; NA: not available data.
