# Supplementary material for: Holobiont dysbiosis or acclimatation? Shift in the microbial taxonomic diversity and functional composition of a cosmopolitan sponge subjected to chronic pollution in a Patagonian bay
Source: PeerJ. 2024 Aug 21;12:e17707. doi: 10.7717/peerj.17707 (PMC11344537; doi:10.7717/peerj.17707)
Supplement: Supplemental Information 9 — * indicate haplotype sequences used in the analyses. CTL, PV, and SAO indicate sites with low, medium, and high pollution level. The description of pollution levels can be found in Table S1. [file peerj-12-17707-s009.docx]

# **Holobiont dysbiosis or acclimatization? Shift in the microbial taxonomic diversity and functional composition of a cosmopolitan sponge subjected to chronic pollution in a Patagonian Bay**

Marianela Gastaldi^1,2^, M. Sabrina Pankey^3^, Guillermo M. Svendsen^1,4*^, Alonso I. Medina^1^, Fausto N. Firstater^1,2^, Maite A. Narvarte^1,2^, Mariana Lozada^5^, Michael P. Lesser^3^

**Supplemental Table S2:** Summary table of the *Hymeniacidon perlevis* sequences used for phylogenetic analyses. * indicate haplotype sequences used in the analyses. CTL, PV and SAO indicate sites with low, medium and high pollution impact.

| nickname/code | GenBank sequences code | Organisms as named in GenBank | gene | R primer | F primer | collection locality | study |
| --- | --- | --- | --- | --- | --- | --- | --- |
| H. perlevis-PV* | MZ297337 | *Hymeniacidon perlevis* | COI | LCOm13 | HCO | SAB | This study |
| H. perlevis-CTL* | MZ297339 | *Hymeniacidon perlevis* | COI | LCOm13 | HCO | SAB | This study |
| H. perlevis-PV* | MZ297338 | *Hymeniacidon perlevis* | COI | LCOm13 | HCO | SAB | This study |
| H. perlevis-CTL | MZ297340 | *Hymeniacidon perlevis* | COI | LCOm13 | HCO | SAB | This study |
| H. perlevis-SAO | MZ297344 | *Hymeniacidon perlevis* | COI | LCOm13 | HCO | SAB | This study |
| H. perlevis-PV | MZ297349 | *Hymeniacidon perlevis* | COI | LCOm13 | HCO | SAB | This study |
| H. perlevis-SAO | MZ297351 | *Hymeniacidon perlevis* | COI | LCOm13 | HCO | SAB | This study |
| H. perlevis-SAO | MZ297353 | *Hymeniacidon perlevis* | COI | LCOm13 | HCO | SAB | This study |
| H. perlevis-SAO | MZ297354 | *Hymeniacidon perlevis* | COI | LCOm13 | HCO | SAB | This study |
| H. perlevis-CTL* | MZ297341 | *Hymeniacidon perlevis* | COI | LCOm13 | HCO | SAB | This study |
| H. perlevis-SAO | MZ297345 | *Hymeniacidon perlevis* | COI | LCOm13 | HCO | SAB | This study |
| H. perlevis-PV | MZ297348 | *Hymeniacidon perlevis* | COI | LCOm13 | HCO | SAB | This study |
| H. perlevis-SAO | MZ297352 | *Hymeniacidon perlevis* | COI | LCOm13 | HCO | SAB | This study |
| H. perlevis-SAO* | MZ297346 | *Hymeniacidon perlevis* | COI | LCOm13 | HCO | SAB | This study |
| H. perlevis-SAO | MZ297347 | *Hymeniacidon perlevis* | COI | LCOm13 | HCO | SAB | This study |
| H. flavia * | EF217335.1 | *Hymeniacidon flavia* | COI |  |  | Korea | Park et al. 2007 |
| H. flavia * | EF217334.1 | *Hymeniacidon flavia* | COI |  |  | Korea | Park et al. 2007 |
| H. flavia | EF217333.1 | *Hymeniacidon flavia* | COI |  |  | Korea | Park et al. 2007 |
| H. heliophila * | EF519630.1 | *Hymeniacidon heliophila* | COI |  |  | Caribbean | Erpenbeck et al. 2008 |
| H. heliophila | EF519631 | *Hymeniacidon heliophila* | COI |  |  | Caribbean | Erpenbeck et al. 2008 |
| H. perlevis * | MG885805.1 | *Hymeniacidon perlevis* | COI |  |  | SAB | Gastaldi et al. 2018 |
| H. perlevis | MG885804.1 | *Hymeniacidon perlevis* | COI |  |  | SAB | Gastaldi et al. 2018 |
| H. perlevis | MG885802.1 | *Hymeniacidon perlevis* | COI |  |  | SAB | Gastaldi et al. 2018 |
| H. perlevis | MG885803.1 | *Hymeniacidon perlevis* | COI |  |  | SAB | Gastaldi et al. 2018 |
| H. perlevis * | HQ829181.1 | *Hymeniacidon sinapium* | COI |  |  | China | Cao H, Cao X, Xue S and Zhang W_direct submission |
| H. perlevis * | NH035986.1 | *Hymeniacidon sinapium* | COI |  |  | China | Cao H, Cao X, Xue S and Zhang W_direct submission |
| H. perlevis * | EF217332.1 | *Hymeniacidon sinapium* | COI |  |  | China | Cao H, Cao X, Xue S and Zhang W_direct submission |
| H. perlevis * | EF217330.1 | *Hymeniacidon sinapium* | COI |  |  | China | Cao H, Cao X, Xue S and Zhang W_direct submission |
| H. perlevis-SAO* | MZ298279 | *Hymeniacidon perlevis* | 18S | 1080R | 18saFm13 | SAB | This study |
| H. perlevis-PV* | MZ298269 | *Hymeniacidon perlevis* | 18S | 1080R | 18saFm13 | SAB | This study |
| H. perlevis-CTL | MZ298271 | *Hymeniacidon perlevis* | 18S | 1080R | 18saFm13 | SAB | This study |
| H. perlevis-SAO | MZ298276 | *Hymeniacidon perlevis* | 18S | 1080R | 18saFm13 | SAB | This study |
| H. perlevis-SAO | MZ298283 | *Hymeniacidon perlevis* | 18S | 1080R | 18saFm13 | SAB | This study |
| H. perlevis-PV* | MZ298268 | *Hymeniacidon perlevis* | 18S | 1080R | 18saFm13 | SAB | This study |
| H. perlevis-CTL | MZ298273 | *Hymeniacidon perlevis* | 18S | 1080R | 18saFm13 | SAB | This study |
| H. perlevis-CTL | MZ298275 | *Hymeniacidon perlevis* | 18S | 1080R | 18saFm13 | SAB | This study |
| H. perlevis-SAO | MZ298277 | *Hymeniacidon perlevis* | 18S | 1080R | 18saFm13 | SAB | This study |
| H. perlevis-SAO | MZ298280 | *Hymeniacidon perlevis* | 18S | 1080R | 18saFm13 | SAB | This study |
| H. perlevis-PV | MZ298281 | *Hymeniacidon perlevis* | 18S | 1080R | 18saFm13 | SAB | This study |
| H. perlevis-PV* | MZ298270 | *Hymeniacidon perlevis* | 18S | 1080R | 18saFm13 | SAB | This study |
| H. perlevis-CTL | MZ298272 | *Hymeniacidon perlevis* | 18S | 1080R | 18saFm13 | SAB | This study |
| H. perlevis-CTL | MZ298274 | *Hymeniacidon perlevis* | 18S | 1080R | 18saFm13 | SAB | This study |
| H. perlevis-SAO | MZ298278 | *Hymeniacidon perlevis* | 18S | 1080R | 18saFm13 | SAB | This study |
| H. perlevis-PV | MZ298282 | *Hymeniacidon perlevis* | 18S | 1080R | 18saFm13 | SAB | This study |
| H. perlevis-SAO | MZ298284 | *Hymeniacidon perlevis* | 18S | 1080R | 18saFm13 | SAB | This study |
| H. perlevis-SAO | MZ298285 | *Hymeniacidon perlevis* | 18S | 1080R | 18saFm13 | SAB | This study |
| H. perlevis-SAO | MZ298286 | *Hymeniacidon perlevis* | 18S | 1080R | 18saFm13 | SAB | This study |
| H. perlevis-SAO | MZ298287 | *Hymeniacidon perlevis* | 18S | 1080R | 18saFm13 | SAB | This study |
| H. perlevis * | MG888753.1 | *Hymeniacidon perlevis* | 18S |  |  | SAB | Gastaldi et al. 2018 |
| H. perlevis * | MG888750.1 | *Hymeniacidon perlevis* | 18S |  |  | SAB | Gastaldi et al. 2018 |
| H. perlevis * | MG888745.1 | *Hymeniacidon perlevis* | 18S |  |  | SAB | Gastaldi et al. 2018 |
| H. perlevis * | MG888744.1 | *Hymeniacidon perlevis* | 18S |  |  | SAB | Gastaldi et al. 2018 |
| H. perlevis * | MG888743.1 | *Hymeniacidon perlevis* | 18S |  |  | SAB | Gastaldi et al. 2018 |
| H. perlevis * | MG888742.1 | *Hymeniacidon perlevis* | 18S |  |  | SAB | Gastaldi et al. 2018 |
| H. perlevis * | MG888741.1 | *Hymeniacidon perlevis* | 18S |  |  | SAB | Gastaldi et al. 2018 |
| H. perlevis * | MG888738.1 | *Hymeniacidon perlevis* | 18S |  |  | SAB | Gastaldi et al. 2018 |
| H. perlevis * | MG888736.1 | *Hymeniacidon perlevis* | 18S |  |  | SAB | Gastaldi et al. 2018 |
| H. perlevis | MG888752.1 | *Hymeniacidon perlevis* | 18S |  |  | SAB | Gastaldi et al. 2018 |
| H. perlevis | MG888751.1 | *Hymeniacidon perlevis* | 18S |  |  | SAB | Gastaldi et al. 2018 |
| H. perlevis | MG888749.1 | *Hymeniacidon perlevis* | 18S |  |  | SAB | Gastaldi et al. 2018 |
| H. perlevis | MG888747.1 | *Hymeniacidon perlevis* | 18S |  |  | SAB | Gastaldi et al. 2018 |
| H. perlevis | MG888746.1 | *Hymeniacidon perlevis* | 18S |  |  | SAB | Gastaldi et al. 2018 |
| H. perlevis | MG888748.1 | *Hymeniacidon perlevis* | 18S |  |  | SAB | Gastaldi et al. 2018 |
| H. perlevis | MG888740.1 | *Hymeniacidon perlevis* | 18S |  |  | SAB | Gastaldi et al. 2018 |
| H. perlevis | MG888739.1 | *Hymeniacidon perlevis* | 18S |  |  | SAB | Gastaldi et al. 2018 |
| H. perlevis | MG888737.1 | *Hymeniacidon perlevis* | 18S |  |  | SAB | Gastaldi et al. 2018 |
| H. perlevis | MG888735.1 | *Hymeniacidon perlevis* | 18S |  |  | SAB | Gastaldi et al. 2018 |
| Halichondria sp. * | MG888754.1 | *Halichondria sp.* | 18S |  |  | SAB | Gastaldi et al. 2018 |
| H. caerulea * | KC902377.1 | *Hymeniacidon caerulea* | 18S |  |  | Panama | Redmon et al. 2013 |
| H. perlevis * | KC902358.1 | *Hymeniacidon perlevis* | 18S |  |  | Ireland | Redmon et al. 2013 |
| H. perlevis-CTL* | MZ435931 | *Hymeniacidon perlevis* | ITS2 | ITS2R | ITSFm13 | SAB | This study |
| H. perlevis-CTL* | MZ435932 | *Hymeniacidon perlevis* | ITS2 | ITS2R | ITSFm13 | SAB | This study |
| H. perlevis-CTL* | MZ435933 | *Hymeniacidon perlevis* | ITS2 | ITS2R | ITSFm13 | SAB | This study |
| H. perlevis-CTL* | MZ435935 | *Hymeniacidon perlevis* | ITS2 | ITS2R | ITSFm13 | SAB | This study |
| H. perlevis-SAO* | MZ435936 | *Hymeniacidon perlevis* | ITS2 | ITS2R | ITSFm13 | SAB | This study |
| H. perlevis-SAO* | MZ435937 | *Hymeniacidon perlevis* | ITS2 | ITS2R | ITSFm13 | SAB | This study |
| H. perlevis-SAO* | MZ435938 | *Hymeniacidon perlevis* | ITS2 | ITS2R | ITSFm13 | SAB | This study |
| H. perlevis-SAO* | MZ435939 | *Hymeniacidon perlevis* | ITS2 | ITS2R | ITSFm13 | SAB | This study |
| H. perlevis-PV* | MZ435942 | *Hymeniacidon perlevis* | ITS2 | ITS2R | ITSFm13 | SAB | This study |
| H. perlevis-PV* | MZ435930 | *Hymeniacidon perlevis* | ITS2 | ITS2R | ITSFm13 | SAB | This study |
| H. perlevis-SAO* | MZ435943 | *Hymeniacidon perlevis* | ITS2 | ITS2R | ITSFm13 | SAB | This study |
| H. perlevis-SAO* | MZ435944 | *Hymeniacidon perlevis* | ITS2 | ITS2R | ITSFm13 | SAB | This study |
| H. perlevis-SAO* | MZ435947 | *Hymeniacidon perlevis* | ITS2 | ITS2R | ITSFm13 | SAB | This study |
| H. perlevis * | EF217361.1 | *Hymeniacidon sinapium* | ITS2 |  |  | USA | Park et al. 2007 |
| H. perlevis * | EF217360.1 | *Hymeniacidon sinapium* | ITS2 |  |  | Korea | Park et al. 2007 |
| H. perlevis * | EF217359.1 | *Hymeniacidon sinapium* | ITS2 |  |  | Korea | Park et al. 2007 |
| H. perlevis | EF217358.1 | *Hymeniacidon sinapium* | ITS2 |  |  | Korea | Park et al. 2007 |
| H. perlevis | EF217357.1 | *Hymeniacidon sinapium* | ITS2 |  |  | Korea | Park et al. 2007 |
| H. perlevis | EF217356.1 | *Hymeniacidon sinapium* | ITS2 |  |  | Korea | Park et al. 2007 |
| H. perlevis | EF217355.1 | *Hymeniacidon sinapium* | ITS2 |  |  | Korea | Park et al. 2007 |
| H. flavia * | AB373185.1 | *Hymeniacidon flavia* | ITS2 |  |  | Japan | Hoshino et al. 2008 |
| H. flavia * | AB373173.1 | *Hymeniacidon flavia* | ITS2 |  |  | Japan | Hoshino et al. 2008 |
| H. flavia | EF217364.1 | *Hymeniacidon flavia* | ITS2 |  |  | Korea | Park et al. 2007 |
| H. flavia | EF217362.1 | *Hymeniacidon flavia* | ITS2 |  |  | Korea | Park et al. 2007 |
| H. heliophila * | AB373170.1 | *Hymeniacidon heliophila* | ITS2 |  |  | USA | Hoshino et al. 2008 |
