# Supplementary material for: Holobiont dysbiosis or acclimatation? Shift in the microbial taxonomic diversity and functional composition of a cosmopolitan sponge subjected to chronic pollution in a Patagonian bay
Source: PeerJ. 2024 Aug 21;12:e17707. doi: 10.7717/peerj.17707 (PMC11344537; doi:10.7717/peerj.17707)
Supplement: Supplemental Information 10 — ASVs abundance is reported as raw counts, percentage of grouped samples, and percentage of abundance at each site. Low, medium, and high refer to sites with different pollution levels. The description of pollution levels can be found in Table S1. [file peerj-12-17707-s010.docx]

# **Holobiont dysbiosis or acclimatization? Shift in the microbial taxonomic diversity and functional composition of a cosmopolitan sponge subjected to chronic pollution in a Patagonian Bay**

Marianela Gastaldi^1,2^, M. Sabrina Pankey^3^, Guillermo M. Svendsen^1,4*^, Alonso I. Medina^1^, Fausto N. Firstater^1,2^, Maite A. Narvarte^1,2^, Mariana Lozada^5^, Michael P. Lesser^3^

**Supplemental Table S3:** Microbial phyla present in water and *Hymeniacidon perlevis* samples. ASVs abundance is reported as raw counts and percentage of grouped samples and percentage of abundance at each site. Low, medium and high refer to sites with low, medium and high pollution.

| Phylum | Source | #ASV | % abundance | % abundance | | |
| --- | --- | --- | --- | --- | --- | --- |
|  |  |  |  | low | medium | high |
| Pseudomonadota | water | 186367 | 60.10036 | 55.25757 | 61.04477 | 65.94554 |
| Bacteroidiota | water | 69717 | 22.48261 | 20.21536 | 24.56414 | 22.44046 |
| Nanoarchaeaeota | water | 7363 | 2.374449 | 4.214049 | 0.954252 | 1.953184 |
| Verrucomicrobiota | water | 6882 | 2.219334 | 3.041359 | 2.066339 | 1.214768 |
| Euryarchaeota | water | 6345 | 2.04616 | 1.719531 | 2.957779 | 1.003208 |
| Actinomycetota | water | 5384 | 1.736253 | 1.850915 | 2.168609 | 0.827134 |
| Chloroflexota | water | 5179 | 1.670144 | 3.240211 | 0.90352 | 0.555518 |
| Planctomycetota | water | 3308 | 1.066777 | 1.402612 | 0.851177 | 0.915853 |
| Campylobacterota | water | 2995 | 0.965839 | 0.953421 | 0.944589 | 1.020951 |
| Patescibacteria group | water | 2570 | 0.828784 | 1.444335 | 0.348685 | 0.696103 |
| Kiritimatiellota | water | 2459 | 0.792988 | 1.053734 | 0.477529 | 0.926773 |
| Nitrososphaerota | water | 1994 | 0.643033 | 1.149609 | 0.3205 | 0.410837 |
| Cyanobacteriota | water | 1821 | 0.587243 | 0.60188 | 0.710254 | 0.356241 |
| Bacillota | water | 1591 | 0.513072 | 0.783865 | 0.34949 | 0.373985 |
| Acidobacteriota | water | 1122 | 0.361827 | 0.636502 | 0.236751 | 0.151505 |
| Mycoplasmatota | water | 894 | 0.288301 | 0.451854 | 0.166692 | 0.242954 |
| Lentisphaerota | water | 805 | 0.2596 | 0.237024 | 0.236751 | 0.333038 |
| Fusobacteriota | water | 551 | 0.177689 | 0.147363 | 0.174745 | 0.229305 |
| Gemmatimonadota | water | 452 | 0.145763 | 0.247676 | 0.099049 | 0.068245 |
| Spirochaetota | water | 377 | 0.121576 | 0.209504 | 0.063617 | 0.084624 |
| Uncul. sponge symbiont PAUC34f | water | 314 | 0.10126 | 0.202402 | 0.057175 | 0.020474 |
| Fibrobacterota | water | 291 | 0.093843 | 0.181097 | 0.035432 | 0.058691 |
| Nitrospirota | water | 207 | 0.066754 | 0.11718 | 0.050732 | 0.016379 |
| Thermodesulfobacteriota | water | 176 | 0.056757 | 0.122507 | 0.025769 | 0.008189 |
| PVC group | water | 176 | 0.056757 | 0.076345 | 0.052343 | 0.034123 |
| Entotheonellaeota | water | 97 | 0.031281 | 0.051488 | 0.024964 | 0.010919 |
| Candidatus Margulisiibacteriota | water | 83 | 0.026766 | 0.064804 | 0.008053 | 0 |
| Deinococcota | water | 81 | 0.026121 | 0.026632 | 0.024964 | 0.027298 |
| Nitrospinota | water | 80 | 0.025799 | 0.051488 | 0.01369 | 0.006825 |
| AncK6 | water | 67 | 0.021606 | 0.051488 | 0.007247 | 0 |
| Candidatus Poribacteria | water | 65 | 0.020961 | 0.047937 | 0.008858 | 0 |
| Candidatus Dependentiae | water | 50 | 0.016124 | 0.030183 | 0.002416 | 0.017744 |
| Chlamydiota | water | 49 | 0.015802 | 0.039948 | 0.001611 | 0.00273 |
| Candidatus Latescibacteria | water | 36 | 0.011609 | 0.008877 | 0.011274 | 0.016379 |
| Elusimicrobiota | water | 35 | 0.011287 | 0.018642 | 0.009663 | 0.00273 |
| Thermoproteota | water | 25 | 0.008062 | 0.009765 | 0.005637 | 0.009554 |
| Cloacimonetes | water | 13 | 0.004192 | 0.003551 | 0.007247 | 0 |
| Candidatus Marinimicrobia | water | 10 | 0.003225 | 0.004439 | 0.004026 | 0 |
| Candidatus Bipolaricaulota | water | 9 | 0.002902 | 0 | 0.004026 | 0.00546 |
| Aegiribacteria | water | 8 | 0.00258 | 0 | 0.003221 | 0.00546 |
| Halanaerobiaeota | water | 8 | 0.00258 | 0.004439 | 0.002416 | 0 |
| Calditrichota | water | 5 | 0.001612 | 0.004439 | 0 | 0 |
| Candidatus Hydrogenedentes | water | 5 | 0.001612 | 0.004439 | 0 | 0 |
| candidate division WS2 | water | 5 | 0.001612 | 0.004439 | 0 | 0 |
| Candidate division Zixibacteria | water | 5 | 0.001612 | 0 | 0 | 0.006825 |
| Synergistota | water | 4 | 0.00129 | 0.003551 | 0 | 0 |
| candidate division FCPU426 | water | 3 | 0.000967 | 0.002663 | 0 | 0 |
| Candidatus Hydrothermarchaeota | water | 3 | 0.000967 | 0.002663 | 0 | 0 |
| Candidatus Saganbacteria | water | 3 | 0.000967 | 0.002663 | 0 | 0 |
| Candidatus Altarchaeota | water | 2 | 0.000645 | 0.001775 | 0 | 0 |
| Candidatus Moduliflexota | water | 2 | 0.000645 | 0.001775 | 0 | 0 |
| Pseudomonadota | sponge | 447579 | 78.1844 | 87.85614 | 72.52198 | 75.98403 |
| Bacteroidiota | sponge | 72186 | 12.60037 | 6.864783 | 17.66819 | 12.91868 |
| Cyanobacteriota | sponge | 10613 | 1.852544 | 1.611595 | 3.243709 | 1.234675 |
| Planctomycetota | sponge | 9452 | 1.649886 | 1.304268 | 1.27071 | 2.035808 |
| Nitrososphaerota | sponge | 6332 | 0.816986 | 0.283477 | 0.879878 | 1.660648 |
| Actinomycetota | sponge | 5548 | 0.927139 | 0.369338 | 0.801173 | 1.374864 |
| Verrucomicrobiota | sponge | 5394 | 0.941545 | 0.669851 | 1.263984 | 0.912492 |
| Chloroflexota | sponge | 4156 | 0.725447 | 0.083817 | 0.242841 | 1.323329 |
| Acidobacteriota | sponge | 2940 | 0.513189 | 0.216697 | 0.193735 | 0.841136 |
| Spirochaetota | sponge | 2826 | 0.49329 | 0.214652 | 1.307708 | 0.204338 |
| Nitrospirota | sponge | 1647 | 0.287491 | 0.007496 | 0.07265 | 0.550666 |
| Nitrospinota | sponge | 692 | 0.120791 | 0.006814 | 0.028253 | 0.230646 |
| Thermodesulfobacteriota | sponge | 605 | 0.105605 | 0.090631 | 0.051124 | 0.142712 |
| Candidatus Margulisiibacteriota | sponge | 578 | 0.100892 | 0.046338 | 0.217279 | 0.067392 |
| Gemmatimonadota | sponge | 471 | 0.082215 | 0.039523 | 0.043725 | 0.125414 |
| Campylobacterota | sponge | 323 | 0.056381 | 0.098808 | 0.042379 | 0.041444 |
| Calditrichota | sponge | 312 | 0.054461 | 0.003407 | 0 | 0.110638 |
| Lentisphaerota | sponge | 176 | 0.030722 | 0.003407 | 0.004036 | 0.059463 |
| Kiritimatiellota | sponge | 175 | 0.030547 | 0.025895 | 0.020181 | 0.038561 |
| Nanoarchaeaeota | sponge | 161 | 0.028103 | 0.067462 | 0.013454 | 0.015136 |
| Chlamydiota | sponge | 146 | 0.025485 | 0.03816 | 0.008745 | 0.02775 |
| Bacillota | sponge | 141 | 0.024612 | 0.020443 | 0.012108 | 0.033516 |
| Deinococcota | sponge | 91 | 0.015884 | 0 | 0.016145 | 0.024146 |
| Euryarchaeota | sponge | 79 | 0.01379 | 0.01908 | 0.026908 | 0.003964 |
| Patescibacteria group | sponge | 65 | 0.011346 | 0.024532 | 0.008072 | 0.006127 |
| Entotheonellaeota | sponge | 57 | 0.00995 | 0.005451 | 0.01009 | 0.012253 |
| Fusobacteriota | sponge | 39 | 0.006808 | 0.001363 | 0.014799 | 0.005406 |
| Candidatus Latescibacteria | sponge | 32 | 0.005586 | 0.004089 | 0.006054 | 0.006127 |
| Candidatus Dependentiae | sponge | 23 | 0.004015 | 0.008177 | 0.002691 | 0.002523 |
| PVC group | sponge | 15 | 0.002618 | 0.003407 | 0.003363 | 0.001802 |
| Uncul. sponge symbiont PAUC34f | sponge | 11 | 0.00192 | 0.003407 | 0.002018 | 0.001081 |
| Mycoplasmatota | sponge | 5 | 0.000873 | 0.001363 | 0 | 0.001081 |
| Candidatus Hydrogenedentes | sponge | 4 | 0.000698 | 0 | 0 | 0.001442 |
| Vulcanimicrobiota | sponge | 4 | 0.000698 | 0.002726 | 0 | 0 |
| Elusimicrobiota | sponge | 3 | 0.000524 | 0 | 0.002018 | 0 |
| Synergistota | sponge | 3 | 0.000524 | 0.002044 | 0 | 0 |
| Candidatus Bipolaricaulota | sponge | 2 | 0.000349 | 0.001363 | 0 | 0 |
| Candidatus Sumerlaeota | sponge | 2 | 0.000349 | 0 | 0 | 0.000721 |
