# Supplementary material for: Holobiont dysbiosis or acclimatation? Shift in the microbial taxonomic diversity and functional composition of a cosmopolitan sponge subjected to chronic pollution in a Patagonian bay
Source: PeerJ. 2024 Aug 21;12:e17707. doi: 10.7717/peerj.17707 (PMC11344537; doi:10.7717/peerj.17707)
Supplement: Supplemental Information 12 — Supplementary information on ITS2 amplification and sequencing, 18S, COI, 16S amplifications and pipeline, and the statistical approach used in this study. [file peerj-12-17707-s012.docx]

# **Holobiont dysbiosis or acclimatization? Shift in the microbial taxonomic diversity and functional composition of a cosmopolitan sponge subjected to chronic pollution in a Patagonian Bay**

Marianela Gastaldi^1,2^, M Sabrina Pankey^3^, Guillermo Svendsen^1,4*^, Alonso Medina^1^, Fausto Firstater^1,2^, Maite Narvarte^1,2^, Mariana Lozada^5^, Michael Lesser^3^

**ITS2, 18S, COI’s amplification and sequencing details**

Polymerase chain reaction (PCR) amplifications were conducted with a total reaction volume of 10 µL which included 0.5 µL of each primer (10 µmol), 5 µL of NEB OneTaq (New England BioLabs Inc.), 2 µL of DNA or molecular water (the latter used as a control). Controls were included to test for contamination in all reaction mixtures. The thermocycler program consisted of an initial denaturation at 94 ºC for 5 min; 35 cycles of 95 ºC for 20 s, 50 ºC for 30 s, and 72 ºC for 60 s; and a final extension at 72 ºC for 5 min. The PCR products were then electrophoresed on a 1% agarose gel. Positive amplicons were incubated at 37˚C with exonuclease I and shrimp alkaline phosphatase (New England Biolabs) prior to Sanger sequencing (Eurofins Genomics). Sponge sequence’s reads were aligned with MAFFT (Katoh et al., 2005) together with *Hymeniacidon* spp. accessions available from GenBank (**Table S2**). Haplotypes were selected for the analyses using DNA Collapser online software (**Table S2**). The phylogenetic reconstruction was carried out through the Maximum Likelihood and Bayesian methods, using Jukes-Cantor model, after testing for the best model fitting with JModelTest software (Darriba et al., 2012).

**16S amplifications and pipeline details.**

Amplifications were performed in a 25 μL reaction volume with 12.5 μL AmpliTaq Gold 360 Master Mix (Applied Biosystems), 1.0 μL GC-enhancer, 0.5 μL of a 10 μM stock of each primer, 2.0 μL of DNA template (40-60 ng/reaction) and 8.5 μL nuclease free water (Integrated DNA Technologies, Coralville, Iowa). Reactions were performed using the following protocol: initial denaturation for 10 min at 95ºC, 30 cycles of 95ºC for 45 s, 50ºC for 60 s, and 72ºC for 90 s, followed by a 10 min extension at 72ºC. The PCR products were then electrophoresed on a 1% agarose gel. The 16S PCR amplicons containing Fluidigm linkers were sequenced on an Illumina MiniSeq System using the Mid-Output reagent kit (2 x 150 bp reads, ~20-40,000 reads per sample) at the University of Illinois at Chicago (UIC) Research Resources Center’s Sequencing Core. Amplicon sequence variants (ASVs) were inferred and tabulated across samples using DADA2 v1.14 (Callahan et al., 2016). Briefly, raw reads were trimmed off the initial 20bp to remove residual primer and then truncated beyond the first instance of quality scores below 3 (truncQ = 2). The maximum expected error during denoising (maxEE) was 2 and 5 for forward and reverse reads, respectively. The error model was built from the first 100M bases and inspected using ‘plotErrors’. Denoised reads were then merged and chimeric contigs discarded using mergePairs and removeBimeraDenovo, respectively. Taxonomic ranks were assigned to the inferred ASVs using the SILVA ribosomal reference database release 132 (Pruesse et al., 2007), using DADA2 function ‘assignTaxonomy’. Samples with fewer than 1,000 counts and singleton counts and ASVs assigned to the Order “Chloroplast” by SILVA taxonomy were discarded for further analyses.

**Statistical approach**

To evaluate the differences in the microbial α-diversity indicators Observed richness (S) wascalculated with estimate_richness() in *vegan* package (Oksanen et al., 2018) and Simpson evenness was calculated as the Inverse of Simpson/Observed richness (Magurran & McGill, 2011). Simpson index is one of the most meaningful and robust measures that captures the variance of the species abundance distribution, and Simpson evenness is considered a pure measure of evenness (Magurran, 2004) and ranges from 0 to 1. The differences in the α-diversity indices between sources (sponges and seawater) and sites (low, medium, and high pollution), were tested using linear models (two-ways ANOVA) and *a posteriori* Tukey tests, using lm() function from *stats* (R Core Team 2020) and HSD.test() from *agricolae* (De Mendiburu & Simon, 2015).

To evaluate the differences in the microbial β-diversity indicators we constructed Jaccard dissimilarity matrices and compared them conducting permutational multivariate ANOVA (PERMANOVA) with source and site as fixed factors. In the case the interaction term was significant, we test the levels of source within the levels of site (Anderson, 2015). Additionally, we compared the sponge-water dissimilarity among sites using univariate ANOVA, after testing the assumptions. To do so, we used ordinate(), plot_ordination(), metaMDS(), lm(), leveneTest(), shapiro.test(), HSD.test(), adonis2() from *pairwiseAdonis* (Martinez Arbizu, 2020), *phyloseq* (McMurdie & Holmes, 2013), *car* (Fox & Weisberg, 2019), *stats*, *agricolae*, and *vegan* packages. To ensure that significant PERMANOVA results were due to centroid differences and not to an unequal dispersion of variability among groups, multivariate homogeneity of groups dispersions analyses were conducted using betadisper(). Posthoc tests for multivariate pairwise comparisons were performed with pairwise.adonis2().

To assess the ratio of common (among sponges) to opportunistic microbes we analyzed the exclusive ASVs that resulted from the edgeR analysis. Those sequences were searched against NCBI nonredundant nucleotide (nr) database using BLASTN and looked for the isolation source of the first five matching sequences. ASVs were considered “common to other sponge species” if at least one of the five matches explicitly stated a marine sponge species as a host or isolation source, while if no sponge species was found as isolation source, the ASV was considered “opportunistic”. E-value was used as a standard metric to filter the sequences. Sequences with E-value higher than 1e-05 were discarded for further analysis, since it indicates low confidence to infer homologous relationship between the sequences.

To assess the variation in the functional profile, and to identify those functions that were differentially expressed in the sponges exposed to contrasting pollution histories, we used the software PICRUSt2 (Douglas et al., 2020) wrapped into QIIME2. PICRUSt2 infers the gene families likely to be present in a sample, based on the genomic information publicly available for the taxonomic assignment of each ASV. Metabolic functions were predicted out of 16S rRNA gene amplicon sequence data using KEGG Orthology (KO) terms and pathways (Kanehisa & Goto, 2000). We used ggpicrust2 (Yang et al., 2023) to examine functional differences in the microbial profiles among sponges from the different sites.

**References**

Anderson MJ. 2015. Workshop on multivariate analysis of complex experimental designs using the PERMANOVA+ add-on to PRIMER v7. PRIMER-E Ltd. In: Plymouth, UK.

Callahan BJ, McMurdie PJ, Rosen MJ, Han AW, Johnson AJA, Holmes SP. 2016. DADA2: High-resolution sample inference from Illumina amplicon data. *Nature methods* 13:581–583.

Darriba D, Taboada GL, Doallo R, Posada D. 2012. jModelTest 2: more models, new heuristics and parallel computing. *Nature methods* 9:772–772.

De Mendiburu F, Simon R. 2015. *Agricolae-Ten years of an open source statistical tool for experiments in breeding, agriculture and biology*. PeerJ PrePrints 3:e1404v1 https://doi.org/10.7287/peerj.preprints.1404v11.

Douglas GM, Maffei VJ, Zaneveld JR, Yurgel SN, Brown JR, Taylor CM, Huttenhower C, Langille MG. 2020. PICRUSt2 for prediction of metagenome functions. *Nature biotechnology* 38:685–688.

Fox J, Weisberg S. 2019. Using car functions in other functions. *CRAN R*. 7 pp. https://scholar.google.com/scholar_lookup?title=Using%20car%20functions%20in%20other%20functions&publication_year=2015&author=J.%20Fox&author=S.%20Weisberg

Kanehisa M, Goto S. 2000. KEGG: kyoto encyclopedia of genes and genomes. *Nucleic acids research* 28:27–30.

Katoh K, Kuma K, Toh H, Miyata T. 2005. MAFFT version 5: improvement in accuracy of multiple sequence alignment. *Nucleic acids research* 33:511–518.

Magurran AE. 2004. *Measuring biological diversity.* Oxford: Blackwell Publishing.

Magurran AE, McGill BJ. 2011. *Biological diversity: frontiers in measurement and assessment*. Oxford University Press.

Martinez Arbizu P. 2020. pairwiseAdonis: Pairwise multilevel comparison using adonis. https://github.com/%20pmartinezarbizu/pairwiseAdonis

McMurdie PJ, Holmes S. 2013. phyloseq: an R package for reproducible interactive analysis and graphics of microbiome census data. *PloS one* 8.

Oksanen J, Blanchet FG, Kindt R, Legendre P, Minchin PR, O’hara RB, Simpson GL, Solymos P, Stevens MHH, Wagner H. 2018. Package ‘vegan’community ecology package. *See https://cran. r-project. org/web/packages/vegan/index. html*.

Pruesse E, Quast C, Knittel K, Fuchs BM, Ludwig W, Peplies J, Glöckner FO. 2007. SILVA: a comprehensive online resource for quality checked and aligned ribosomal RNA sequence data compatible with ARB. *Nucleic acids research* 35:7188–7196.

Yang C, Mai J, Cao X, Burberry A, Cominelli F, Zhang L. 2023. ggpicrust2: an R package for PICRUSt2 predicted functional profile analysis and visualization. *Bioinformatics* 39:btad470. DOI: 10.1093/bioinformatics/btad470.
